# Differential Circulating miRNA Responses to PM Exposure in Healthy and Diabetes Mellitus Patients: Implications for Lung Cancer Susceptibility

**DOI:** 10.3390/ijms27020613

**Published:** 2026-01-07

**Authors:** Moe Thi Thi Han, Nichakorn Satitpornbunpot, Naoomi Tominaga, Saranta Freeouf, Khanittha Punturee, Chidchamai Kewchareonwong, Busayamas Chewaskulyong, Ganjana Lertmemongkolchai, Ratchada Cressey

**Affiliations:** 1 Department of Medical Technology, Faculty of Associated Medical Sciences, Chiang Mai University, Chiang Mai 50200, Thailand; moethithi_han@cmu.ac.th (M.T.T.H.); nichakorn_sat@cmu.ac.th (N.S.); srtfreeouf@gmail.com (S.F.); khanittha.taneyhill@cmu.ac.th (K.P.); chidchamai.k@cmu.ac.th (C.K.); ganjana.l@cmu.ac.th (G.L.); 2 Division of Clinical Laboratory Sciences, Department of Nursing and Laboratory Science, Graduate School of Medicine, Yamaguchi University, 1-1-1 Minami-kogushi, Ube 755-8505, Japan; ntominag@yamaguchi-u.ac.jp; 3 Department of Internal Medicine, Faculty of Medicine, Chiang Mai University, Chiang Mai 50200, Thailand; bchewask@gmail.com

**Keywords:** particulate matter, air pollution, circulating microRNA, miR-542-3p, miR-29a-3p, diabetes mellitus, exosomes, interleukin-8, lung cancer, biomarkers

## Abstract

Seasonal biomass-burning haze in Northern Thailand produces sharp fluctuations in ambient fine particulate matter (PM), posing heightened health risks, particularly for individuals with diabetes mellitus (DM). To identify PM-responsive biomarkers and assess whether metabolic status modifies these responses, we first performed small RNA sequencing in a discovery cohort using plasma samples collected during low- and high-PM periods. Thirteen circulating microRNAs (miRNAs) were differentially expressed, including reduced miR-542-3p and elevated miR-29a-3p, novelmiR-203, and novelmiR-754, with predicted targets enriched in immune and endoplasmic-reticulum stress pathways. These four miRNAs were quantified by RT-qPCR in a longitudinal cohort of adults with (n = 28) and without DM (n = 29) sampled at three PM-defined timepoints across one full haze cycle. In non-DM individuals, miR-542-3p decreased at peak exposure while miR-29a-3p and novelmiR-203 increased, with values returning toward baseline at re-exposure. DM participants showed altered baseline levels and attenuated or reversed seasonal changes. Plasma IL-8 rose markedly at peak PM in both groups, mirroring exosome concentration increases measured by NTA, indicating a transient systemic inflammatory response. In an independent clinical cohort, only miR-542-3p differed significantly between lung-cancer patients and healthy controls. These findings indicate that PM exposure reconfigures circulating miRNA, exosomal, and cytokine profiles, and that DM modifies these responses, highlighting miR-542-3p and miR-29a-3p as environmentally responsive and disease-relevant biomarker candidates.

## 1. Introduction

Northern Thailand’s seasonal haze—primarily driven by open biomass burning of crop residues, forest and edge fires, and local waste burning during the dry season—recurs each year from January to May, with March peaks that trap smoke in mountain basins [1,2]. During these episodes, PM2.5 (particulate matter with an aerodynamic diameter ≤ 2.5 µm) commonly reaches concentrations exceeding 100 µg/m^3^ and PM10 (≤10 µm) can approach 300 µg/m^3^, with annual PM10 mean values around 43–61 µg/m^3^, substantially exceeding World Health Organization (WHO) guideline values [3]. These high-percentile haze days are major contributors to acute healthcare utilization and are associated with increased long-term respiratory and cardiovascular mortality, as well as elevated lung cancer risk [4]. Mechanistically, particulate exposure induces oxidative stress and pattern-recognition receptor signaling, leading to robust upregulation of pro-inflammatory mediators such as interleukin-8 (IL-8) and promoting neutrophilic airway inflammation with systemic consequences [5,6]. While established protein markers such as IL-6 and CRP reflect inflammation status [7,8], they lack exposure-specificity and are strongly influenced by chronic comorbidities, most notably diabetes mellitus (DM).

People living with diabetes experience a heightened cardiometabolic and pulmonary vulnerability, and accumulating evidence indicates heightened susceptibility to particulate air pollution. In adults with diabetes, short-term PM2.5 exposure increases expression of antigen-presentation and costimulatory receptors on circulating monocytes (CD80, CD40, CD86, HLA-DR, CD23) [9] and elicits distinct and amplified respiratory inflammatory responses compared with people with prediabetes individuals [10]. Experimental models of type 2 diabetes further demonstrate exaggerated metabolic and inflammatory consequences following inhalational PM2.5 exposure, including worsened insulin resistance, visceral adipose inflammation, altered adipokines profile and accelerated diabetogenesis under high-fat diet conditions [11,12]. Epidemiologic studies have also link long-term PM2.5 exposure to incident type 2 diabetes [13] and to worsened glycemic control among patients with established disease [12], with emerging data implicating the gut microbiome as a partial mediator [14]. Despite this converging evidence, sensitive molecular tools capable of capturing the incremental inflammatory and metabolic burden of polluted air in dysglycemic populations remained limited.

Circulating microRNAs (miRNAs) represent attractive translational biomarkers because they are stable in blood, protected within extracellular vesicles, and functionally involved in pathways regulating stress responses, inflammation, metabolism, and cancer. Experimental and population-based studies have demonstrated that exposure to particulate air pollution is associated with systematic alterations in circulating miRNA expression profiles, particularly among miRNAs involved in inflammatory signaling, oxidative stress, and cardiopulmonary disease pathways [15,16]. Accordingly, a validated miRNA panel could enable an exposure-responsive and susceptibility-aware assessment from a single blood sample, thereby bridging environmental exposure with clinically actionable risk stratification, especially in populations with diabetes. However, longitudinal human studies that integrate PM exposure, circulating miRNAs, and systemic clinical markers—while directly comparing individuals with and without diabetes—remain scarce.

To address this gap, we employed a stepwise study design. We first performed unbiased small RNA sequencing to identify circulating miRNAs responsive to seasonal PM exposure. From this discovery phase, miR-542-3p, miR-29a-3p, novelmiR-203, and novelmiR-754 were prioritized based on statistical significance and predicted involvement in inflammatory, stress-response, and cancer-related pathways. We then conducted longitudinal RT-qPCR validation across pre-exposure, peak-haze, and re-exposure timepoints in adults with and without diabetes, and integrated miRNA profiles with systemic markers (IL-6, CRP, HbA1c, and fasting glucose) to link molecular alterations with clinically relevant phenotypes. Our objective was to develop and validate a circulating miRNA signature of ambient PM exposure that complements conventional inflammatory markers and informs risk assessment in both diabetic and non-diabetic adults.

## 2. Results

### 2.1. Differential Expression of miRNAs and Functional Enrichment Analysis in Low- vs. High-PM Samples

**Figure 1:**
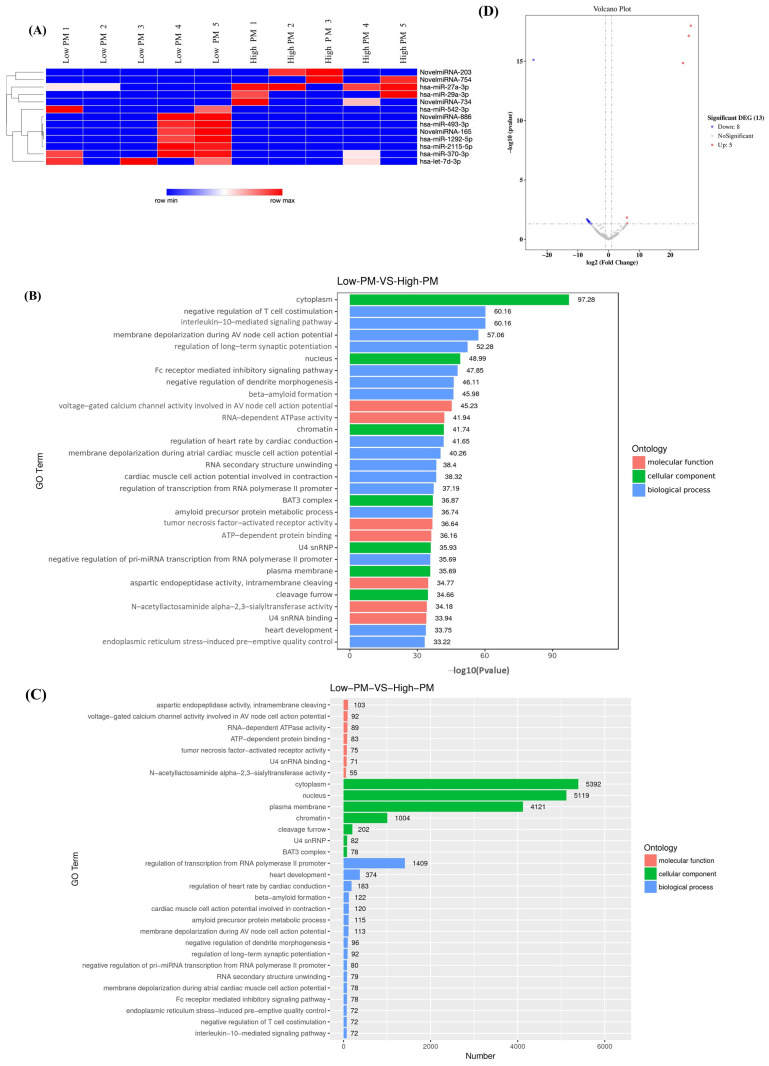
Differentially expressed circulating miRNAs and GO enrichment between low- and high-PM groups. (**A**) Unsupervised hierarchical clustering heatmap of differentially expressed circulating miRNAs identified by small-RNA sequencing in individuals from low-PM (Low PM1–5) and high-PM (High PM1–5) environments (n = 5 per group). Colors represent z-score–scaled normalized expression values (red, higher; blue, lower). (**B**) GO enrichment of predicted target genes ranked by −log10(*p*), highlighting immune regulation, neuronal signaling, cardiac conduction and ER-stress–related pathways. (**C**) GO terms ranked by number of target genes, with predominant enrichment in cytoplasm, nucleus and plasma membrane, indicating widespread cellular impact. (**D**) Volcano plot showing 13 significantly dysregulated miRNAs (5 upregulated, 8 downregulated) in high- vs. low-PM samples (n = 5 per group), including strong downregulation of miR-542-3p and upregulation of miR-29a-3p, novelmiR-203 and novelmiR-754. The horizontal dashed line indicates the significance threshold (FDR-adjusted *p*-value), and the vertical dashed lines indicate the log_2_ fold-change cutoffs. Grey dots represent miRNAs that did not reach the significance thresholds. Differential expression was assessed using DESeq2 (Wald test) with Benjamini–Hochberg FDR correction.

To explore the effect of particulate matter (PM) exposure on circulating miRNA profiles, we performed unsupervised hierarchical clustering of differentially expressed miRNAs between individuals during low- and high-PM environments (Figure 1A). The resulting heatmap demonstrated a clear separation of samples according to PM level, indicating distinct expression patterns associated with elevated PM exposure. Compared with the low-PM group, the high-PM group showed markedly higher expression of several miRNAs, including hsa-miR-27a-3p, hsa-miR-29a-3p, hsa-miR-493-3p, hsa-miR-2115-5p, and the novel species novelmiR-203, novelmiR-734, and novelmiR-886, whereas lower relative expression was observed for hsa-miR-542-3p (notably reduced across high-PM samples), hsa-miR-1292-5p, hsa-miR-370-3p, and hsa-let-7d-3p. Together, these coordinated shifts indicate that elevated PM exposure is associated with systematic remodeling of circulating miRNA profiles.

To understand the potential biological implications of these miRNA changes, we performed Gene Ontology (GO) enrichment analysis of their predicted target genes. When ranked by statistical significance (−log10 *p*-value), enriched terms (Figure 1B) highlighted pathways related to immune regulation, including negative regulation of T-cell co-stimulation and interleukin-10-mediated signaling pathway, as well as pathways related to neuronal signaling, cardiac conduction, and endoplasmic reticulum (ER) stress. Cellular component enrichment revealed predominant localization to the cytoplasm, nucleus, and plasma membrane, indicating that PM-responsive miRNAs target genes distributed across multiple cellular compartments.

When GO terms were assessed based on the absolute number of genes represented (Figure 1C), the most prominent categories were cytoplasm (5392 genes), nucleus (5119 genes), and plasma membrane (4121 genes). Large gene sets were also associated with transcriptional regulation, heart development, dendritic remodeling, and ER stress responses, reinforcing the concept that PM-induced miRNA alterations exert broad effects on cellular structure and function.

The volcano plot (Figure 1D) identified 13 significantly dysregulated miRNAs, including 8 downregulated and 5 upregulated miRNAs in high-PM samples (Table 1). Among these, miR-542-3p showed strong downregulation, whereas miR-29a-3p, novelmiR-203, and novelmiR-754 were markedly upregulated. These miRNAs may therefore serve as molecular fingerprints of PM exposure, with potential roles in regulating immune pathways, stress adaptation, and cardiovascular or neurological responses.

Together, these analyses indicate that PM exposure substantially reconfigures the circulating miRNA landscape. The dysregulated miRNAs are enriched for pathways involved in immune modulation, neuronal signaling, cardiac conduction, and ER stress, suggesting biologically plausible mechanisms for PM-related health effects. Within this cross-sectional signature, miR-542-3p (reduced in high-PM) and miR-29a-3p together with novelmiR-203 and novelmiR-754 (increased), emerge as priority candidates for downstream longitudinal and clinical analyses. Having defined this high-PM miRNA signature enriched for immune/ER-stress processes, we next asked whether these differences are reproducible within individuals and reversible after the exposure season. To determine whether these differences were reproducible within individuals and reversible after the exposure season, we next profiled the same miRNAs longitudinally across the PM season in an independent cohort.

### 2.2. Longitudinal Changes in Circulating miRNAs with PM Exposure

To validate the small RNA sequencing findings and to determine whether seasonal particulate matter exposure differentially modulates circulating miRNAs in adults with and without diabetes, we quantified four PM-responsive miRNAs—miR-542-3p, miR-29a-3p, novelmiR-203, and novelmiR-754—by RT-qPCR in a longitudinal cohort comprising 29 non-diabetic (non-DM) and 28 diabetic (DM) participants. Baseline demographic and clinical characteristics are summarized in Table 2. The two groups were comparable in age, sex distribution, height, BMI, blood pressure, oxygen saturation, pulse rate, hsCRP, and fasting blood glucose (all *p* > 0.05). HbA1c levels were in the DM group than in the non-DM group (*p* = 0.003), consistent effective glycemic control under anti-diabetic treatment rather than absence of disease. Body weight was modestly higher in the DM group (*p* = 0.031), whereas no other clinical variables differed significantly.

**Figure 2:**
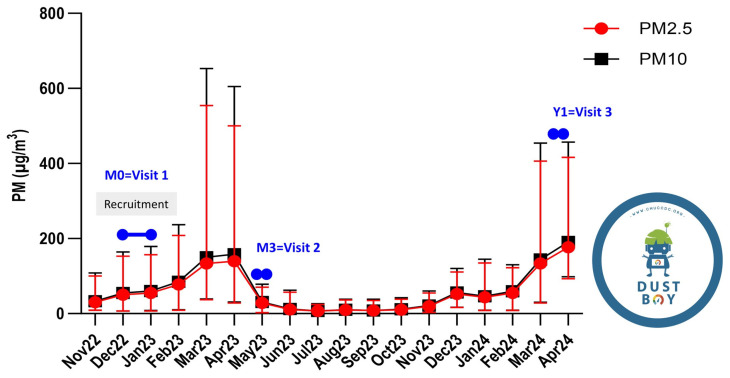
Ambient PM2.5 and PM10 levels during the study period and alignment of participant sampling with the seasonal haze cycle. Monthly mean concentrations of PM2.5 (red circles) and PM10 (black squares) (µg/m^3^) were obtained from the DUSTBOY community air-quality monitoring network (San Na Meng station, ID DBBlack232; https://www.cmducc.org/DBBlack232, accessed on 1 May 2024) from November 2022 to April 2024. Error bars indicate month-to-month variability. Participant blood collections were scheduled relative to the seasonal biomass-burning haze cycle: M0 (Visit 1) during the early rise in PM levels (December 2022–January 2023), M3 (Visit 2) after 3–4 consecutive months of elevated PM (May 2023), and Y1 (Visit 3) at the onset of the subsequent haze season (March 2024). Shaded regions denote the sampling windows used for downstream longitudinal analyses. No statistical testing was applied to ambient PM time-series data.

Participants were sampled at three timepoints spanning the regional PM season in San Na Meng, Chiang Mai (Figure 2). The baseline visit (M0) occurred during the early rise in PM concentrations (December 2022–January 2023; ~150–200 µg/m^3^ PM2.5). The second visit (M3) was conducted after 3–4 consecutive months of sustained high exposure (May 2023), following peaks of 500–600 µg/m^3^ during March–April). The third visit (Y1) corresponded to the onset of the subsequent haze season (March 2024; approximately 400–450 µg/m^3^ PM2.5). These timepoints therefore represent early exposure, post–prolonged exposure, and re-exposure phases, respectively.

**Figure 3:**
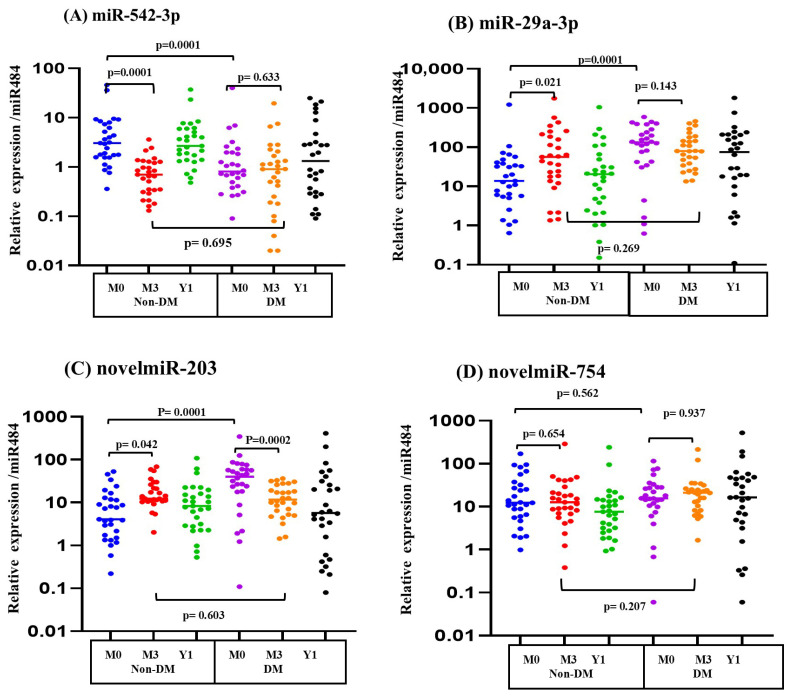
Longitudinal expression of PM-responsive miRNAs in non-diabetic and diabetic adults. Relative plasma miRNA expression (2^−ΔCT^, normalized to miR-484; log_10_ scale where indicated on the y-axis) for (**A**) miR-542-3p, (**B**) miR-29a-3p, (**C**) novelmiR-203, and (**D**) novelmiR-754 in non-DM (n = 29) and DM (n = 28) participants at M0, M3, and Y1. Each colored dot represents an individual participant, with colors indicating sampling timepoints (M0, M3, Y1). Horizontal lines indicate group medians. Non-DM subjects showed a transient decrease in miR-542-3p and increases in miR-29a-3p and novelmiR-203 at M3 with return toward baseline at Y1, whereas DM subjects showed no significant temporal change despite differing baselines. NovelmiR-754 remained stable in both groups. *p*-values were calculated using Wilcoxon signed-rank tests for within-group (paired) comparisons across timepoints and Mann–Whitney U tests for between-group (unpaired) comparisons at each timepoint.

Across this exposure framework, the four circulating miRNAs (normalized to miR-484) demonstrated distinct and miRNA-specific patterns (Figure 3). In non-DM participants, miR-542-3p declined significantly from M0 to M3 and increased again at Y1. In DM participants, miR-542-3p showed no significant temporal variation, although baseline levels at M0 were lower than those observed in non-DM individuals.

For miR-29a-3p, non-DM participants showed a significant rise from M0 to M3 followed by partial normalization at Y1. In contrast, DM participants had higher baseline levels but no significant change across the three timepoints. A similar group-specific pattern was observed for novelmiR-203: non-DM individuals demonstrated an increase at M3, whereas DM participants showed a decrease relative to baseline. NovelmiR-754 did not display significant temporal variation in either group.

Overall, these longitudinal data demonstrate distinct miRNA expression patterns across the PM season in non-DM and DM adults, with group-specific differences in baseline levels and temporal changes.

### 2.3. Comparison of Plasma Exosome Concentration and Size at M0 and M3 in Non-DM and DM

**Figure 4:**
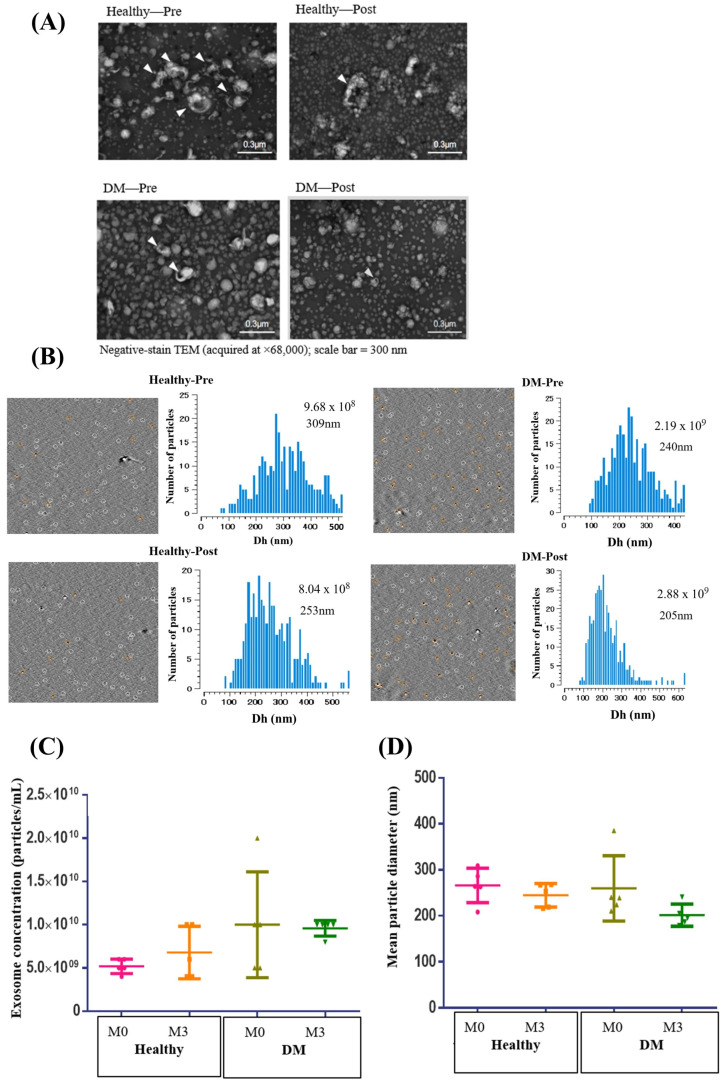
Plasma exosome morphology, concentration and size in non-diabetic (non-DM) and diabetic (DM) participants across the PM season. (**A**) Representative negative-stain TEM images of plasma exosomes from non-DM and DM participants before (M0) and after (M3) the high-PM season, showing typical round/cup-shaped vesicles with intact membranes. White triangles indicate representative exosome-like vesicles (scale bar = 300 nm). (**B**) Representative nanoparticle tracking analysis (NTA) images and size-distribution histograms (diameter in nm) from the same samples, illustrating particle concentrations (particles/mL) on the order of 10^8^–10^9^ particles/mL and modal diameters ~200–300 nm at each timepoint. (**C**) Scatter plots showing exosome concentration (particles/mL) and (**D**) mean particle diameter (nm) measured by NTA in a subset of non-DM and DM participants (n = 5 per group) at M0 and M3. Different colored symbols represent individual participants from different groups and timepoints. Horizontal lines indicate group medians, with error bars representing the interquartile range (IQR). Both groups showed a tendency toward higher exosome counts and slightly smaller mean diameters at M3 compared with M0. Quantitative NTA comparisons were performed in a subset (non-DM n = 5; DM n = 5 per timepoint). Because of the limited sample size, analyses are presented descriptively; statistical testing was not emphasized.

To evaluate whether seasonal PM exposure influences circulating exosome output and whether these patterns differ between non-DM and DM participants, we analyzed plasma exosomes from a subset of non-diabetic (non-DM) and diabetic (DM) participants (n = 5 per group) at baseline (M0) and after 3–4 months of sustained high PM exposure (M3) using transmission electron microscopy (TEM) and nanoparticle tracking analysis (NTA) (Figure 4). TEM confirmed typical exosome morphology in both groups at both timepoints, with round/cup-shaped vesicles and intact membranes, and no obvious qualitative differences between M0 and M3 (Figure 4A).

NTA further demonstrated particle populations predominantly within the expected exosome size range (approximately 100–400 nm), with representative size-distribution histograms showing modal diameters in the ~200–300 nm range (Figure 4B). When summarized across individuals, exosome concentration (particles/mL) showed a tendency to increase from M0 to M3 in both non-DM and DM groups, although there was substantial inter-individual variability (Figure 4C). Mean particle diameter also showed a modest reduction at M3 compared with M0 in both groups, while remaining within the typical size range reported for plasma-derived exosomes (Figure 4D). Because exosome measurements were conducted in a limited subset and only at two timepoints, these NTA findings are presented descriptively.

### 2.4. Plasma IL-8 Across the PM Season

To assess whether seasonal PM exposure corresponded with changes in systemic inflammation, we measured plasma IL-6, IL-8, and TNF-α by ELISA at all three sampling timepoints—pre-season baseline (M0), post–PM season (M3), and the early-season follow-up one year later (Y1). IL-6 and TNF-α were detected at or below the assay limits in most samples and were therefore excluded from analysis. In contrast, IL-8 was consistently detectable in all participants and was selected as the primary inflammatory marker for longitudinal evaluation.

**Figure 5:**
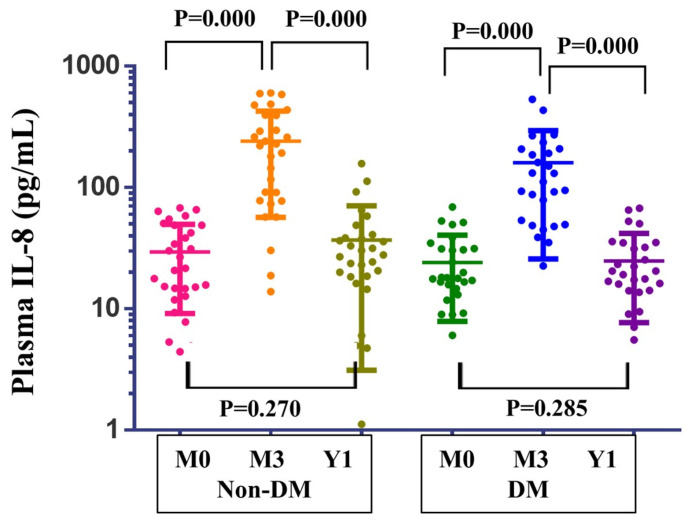
Plasma IL-8 levels across the PM season in non-diabetic and diabetic adults. Plasma IL-8 concentrations (pg/mL; log10 scale) in non-DM (n = 29) and DM (n = 28) participants at pre-season baseline (M0), after 3–4 months of high PM exposure (M3), and at the onset of the following PM season (Y1). Each colored dot represents an individual participant, with colors indicating timepoints (M0, M3, and Y1). Horizontal lines indicate group medians, with error bars representing the interquartile range (IQR). In both groups, IL-8 increased markedly from M0 to M3 and declined again by Y1, with no significant difference between M0 and Y1. *p*-values are from Wilcoxon signed-rank tests (paired) for within-group comparisons across timepoints.

As shown in Figure 5, both non-diabetic (non-DM) and diabetic (DM) participants exhibited a similar temporal pattern of IL-8 expression across the PM season. In both groups, IL-8 levels were lowest at M0, increased markedly at M3 following prolonged high PM exposure, and declined again by the Y1. In non-DM volunteers, IL-8 concentrations at M3 were significantly higher than at M0 (*p* < 0.001) and decreased from M3 to Y1 (*p* < 0.001), with no significant difference between M0 and Y1 (*p* = 0.270). A comparable pattern was observed in the DM group, with IL-8 levels rising from M0 to M3 (*p* < 0.001), falling from M3 to Y1 (*p* < 0.001), and showing no significant difference between M0 and Y1 (*p* = 0.285).

Overall, in both glycemic groups, IL-8 peaked at the M3 timepoint and returned toward pre-season concentrations at Y1, mirroring the three-timepoint sampling structure used throughout the study.

### 2.5. Expression of PM-Responsive miRNAs in Lung-Cancer Patients

To determine whether PM-responsive miRNAs are clinically relevant in lung cancer, we quantified circulating levels of four candidate miRNAs—miR-29a-3p, miR-542-3p, novelmiR-203, and novelmiR-754—in archived serum samples from treatment-naïve lung-cancer patients and age-matched healthy controls. The demographic characteristics of both groups are summarized in Table 3. The cancer cohort (n = 55) and healthy controls (n = 27) were similar in age, while gender distribution showed slightly more males in the cancer group. As expected, smoking status differed markedly, with 34 smokers among cancer patients versus only one smoker in the control group. Most cancer cases were adenocarcinoma (80%), and the majority presented at late clinical stage (Stage III–IV), reflecting the typical pattern of diagnosis in Northern Thailand.

**Figure 6:**
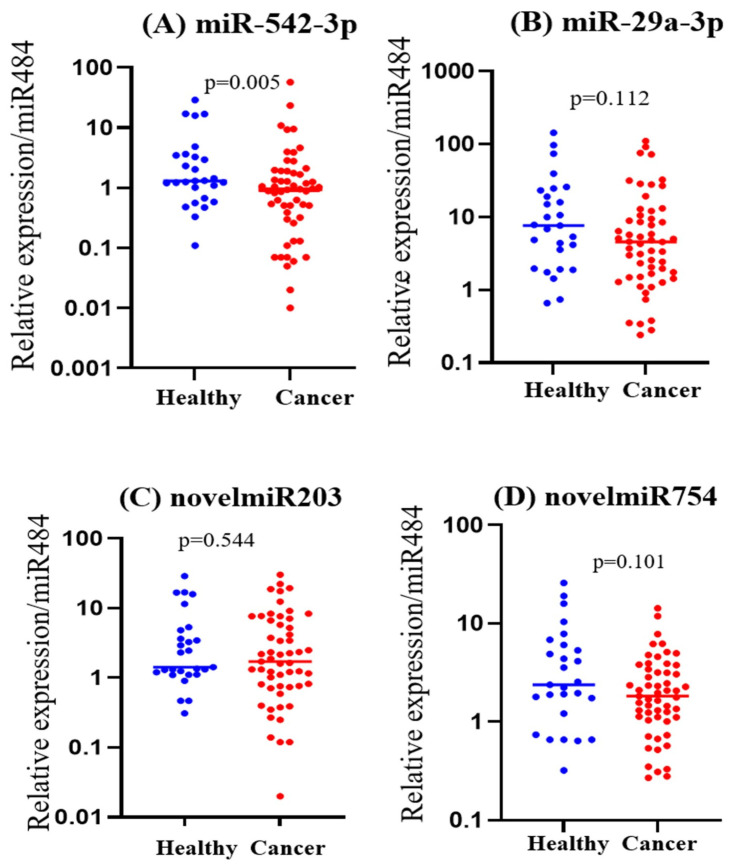
Circulating PM-responsive miRNAs in healthy controls (n = 27) and lung-cancer patients (n = 55). Relative plasma expression of (**A**) miR-542-3p, (**B**) miR-29a-3p, (**C**) novelmiR-203 and (**D**) novelmiR-754, normalized to miR-484, in healthy individuals (n = 27) and treatment-naïve lung-cancer patients (n = 55). Each dot represents one participant; horizontal bars indicate median values (log_10_ scale). miR-542-3p was significantly lower in lung-cancer patients, whereas miR-29a-3p, novelmiR-203 and novelmiR-754 did not differ significantly between groups. *p*-values are from Mann–Whitney U tests.

Among the four candidates, miR-542-3p was significantly lower in lung-cancer patients than in healthy controls (*p* = 0.005) (Figure 6). In contrast, miR-29a-3p (*p* = 0.112), novelmiR-203 (*p* = 0.544), and novelmiR-754 (*p* = 0.101) did not reach statistical significance between groups. However, miR-29a-3p showed downward shift in its distribution in lung-cancer patients compared with healthy individuals, although differences did not reach statistical significance.

## 3. Discussion

In this study, we investigated how ambient particulate matter modifies circulating microRNA profiles in healthy adults and in individuals with type 2 diabetes, and whether PM-responsive miRNAs overlap with expression patterns observed in patients with lung cancer. We applied a stepwise approach: small RNA sequencing in a discovery set to identify PM-responsive circulating miRNAs, RT-qPCR validation of a focused panel in a longitudinal cohort sampled across low exposure, peak haze, and re-exposure (including both non-diabetic and diabetic adults to assess metabolic modification of baseline levels and seasonal responsiveness), and measurement of the same miRNAs in an independent lung-cancer cohort to indicate potential clinical relevance. Our sequencing analysis identified a distinct miRNA signature differentiating low- and high-PM periods, with decreased miR-542-3p and increased miR-29a-3p, novelmiR-203, and novelmiR-754. Predicted target pathways converged on inflammatory signaling, immune regulation, and endoplasmic-reticulum stress, consistent with established mechanisms of PM-induced oxidative and inflammatory injury [17,18,19,20].

Longitudinal analyses further demonstrated that diabetes modifies both baseline miRNA expression and their dynamic responses to environmental stress. In non-diabetic individuals, miR-542-3p exhibited a clear exposure-linked decrease at M3, followed by recovery when air quality improved at Y1. Notably, statistically significant exposure-related changes in circulating miRNAs were observed only at the peak haze timepoint (M3), whereas M0 and Y1 largely reflected baseline and partial recovery states. This pattern suggests that sustained high PM exposure and a lag between ambient peaks and circulating molecular responses are required to elicit measurable miRNA shifts, rather than very short-term fluctuations alone. Diabetic participants, however, began the season with significantly lower miR-542-3p levels at M0—consistent with chronic metabolic inflammation [21,22]—and showed additional suppression in some individuals during peak exposure, though the change did not reach statistical significance. miR-29a-3p and novelmiR-203 also displayed markedly different PM-linked trajectories between the groups. In healthy volunteers, both miRNAs rose significantly at M3 and returned toward baseline at Y1, indicating a coordinated inducible response to PM. In diabetes, however, this adaptive increase was absent: miR-29a-3p failed to rise with PM exposure and novelmiR-203 even decreased from baseline. These divergent patterns are consistent with diabetes-associated inflammatory and metabolic dysregulation, which can impair normal stress-responsive miRNA induction during environmental challenges [23,24]. Collectively, our findings indicate that non-diabetic individuals mount an exposure-dependent miRNA response to PM, whereas diabetes elevates baseline inflammatory tone and disrupts inducibility. By comparison, novelmiR-754 did not exhibit significant seasonal variation in either non-diabetic or diabetic participants in the longitudinal validation cohort. One possible explanation is that novelmiR-754 reflects an early or transient PM-responsive signal, with induction occurring during the initial rise in exposure and diminishing after adaptation to sustained exposure, thereby reducing detectability at later timepoints.

Mechanistically, miR-542-3p is a tumor-suppressive miRNA that inhibits multiple oncogenic pathways activated by PM. It directly targets ILK, OTUB1, AEG-1, cortactin, and ARHGAP1, thereby suppressing AKT, NF-κB, Wnt/β-catenin and c-Src/FAK signaling [25,26,27]. These pathways are central mediators of PM-induced oxidative stress, inflammation and epithelial injury. Our findings are consistent with this model: diabetic participants began the season with lower circulating miR-542-3p levels, likely reflecting chronic inflammatory and oxidative load [21,22], and PM exposure induced further suppression in some individuals. Lung-cancer patients in our cohort also exhibited markedly reduced miR-542-3p levels, reinforcing links between chronic inflammatory stress, impaired tumor-suppressive miRNA regulation, and cancer susceptibility. Consistent with this clinical relevance, hsa-miR-542-3p has been reported among the top discriminative miRNAs measured in whole plasma when comparing lung adenocarcinoma patients with benign granuloma controls, supporting the premise that circulating miR-542-3p is altered in lung disease contexts [28]. While tissue-specific studies demonstrate heterogeneous miR-542-3p expression in diabetes (increased in kidney fibrosis [22] but decreased in cardiac and corneal tissues [21]), the circulating pattern in our cohort—low at baseline in diabetes, decreased further in some individuals during high PM, and lowest in lung-cancer patients—suggests that circulating miR-542-3p reflects systemic inflammatory and oxidative burden.

miR-29a, although also a tumor-suppressive miRNA regulating apoptosis, extracellular-matrix turnover, and the anti-apoptotic factor MCL1 [29,30], showed the opposite acute response. Its increase during high-PM exposure aligns with evidence that circulating miR-29a is elevated in cancers with prominent tissue injury or fibrosis, including colorectal cancer [31,32] and malignant mesothelioma, and summarized as upregulated in meta-analysis [32]. Conversely, miR-29a is reduced in papillary thyroid carcinoma and AML [29,33]. A large prospective cohort associated higher circulating miR-29a with increased risks of cancer and cardiovascular mortality [34]. Together, these studies indicate that circulating miR-29a reflects tissue-injury, remodeling, and vesicle-release processes rather than strictly mirroring intracellular tumor-suppressive functions. Consistent with this interpretation, circulating miR-29a has also been reported in lung cancer–related clinical contexts, including plasma miRNA studies in non-small-cell lung cancer patients undergoing thoracic radiotherapy, supporting its relevance to lung tissue injury and disease-associated remodeling [35,36,37]. Thus, the rise in circulating miR-29a during high PM likely represents a compensatory response to epithelial injury. Its absence in diabetes aligns with impaired tissue-repair and stress-response capacity in metabolically inflamed states.

The opposite directions of miR-542-3p (down) and miR-29a (up) during PM exposure can therefore be understood in the context of their distinct biological roles. miR-542-3p suppresses inflammatory and oncogenic signaling strongly activated by PM, so its reduction reflects a maladaptive response that weakens regulatory control. miR-29a, in contrast, is linked to extracellular-matrix regulation, fibrosis, and injury-repair pathways, which are activated during PM-induced epithelial damage. Despite opposite acute responses, both miRNAs ultimately show reduced levels in lung-cancer patients, consistent with their shared tumor-suppressive functions and suggesting that chronic inflammation and tumor progression eventually suppress both regulators.

The cytokine data provide additional support for PM-linked inflammatory activation. Plasma IL-8 rose sharply at M3 and declined at Y1. IL-8 is one of the most robustly PM-induced cytokines, with controlled exposure studies demonstrating rapid IL-8 release from airway epithelial and monocytic cells; this response is amplified by ozone and attenuated by antioxidant treatment, implicating ROS-dependent induction mechanisms [38]. Beyond being an inflammatory marker, IL-8/CXCR1-CXCR2 signaling promotes epithelial–mesenchymal transition, invasion, cancer-stem cell properties, angiogenesis, and recruitment of protumoral myeloid cells [39]. Exosome measurements provided an additional layer of information on PM-related responses. We observed a numerical increase in circulating exosome concentration at M3 compared with M0 in both healthy and diabetic participants, although these changes did not reach statistical significance (*p* > 0.05), likely reflecting the small number of samples analyzed (n = 5 per group per timepoint). This trend is consistent with previous experimental data showing that PM2.5 and oxidative stress can stimulate extracellular-vesicle release from airway epithelial and immune cells [40]. In contrast, mean particle diameter remained relatively stable over time and within the typical size range reported for small extracellular vesicles (approximately 50–300 nm) [41]. Taken together, these findings suggest that PM exposure may primarily increase the number of circulating vesicles rather than altering their size, supporting the notion that enhanced vesicle release—and its associated miRNA cargo—contributes to PM-related inflammatory and stress-response signaling.

Taken together, these findings support an associative framework in which diabetes is linked to a dysregulated baseline miRNA profile characterized by lower circulating miR-542-3p and altered miR-29a and novelmiR203 levels, while seasonal PM exposure is accompanied by adaptive miRNA and cytokine responses in non-diabetic individuals but attenuated responses in those with metabolic inflammation. Lung-cancer patients in our cohort exhibited further suppression of tumor-suppressive miRNAs, consistent with prior reports in cancer populations. While these observations suggest biological plausibility for a link between PM exposure, metabolic vulnerability, and miRNA dysregulation, causal relationships cannot be inferred from the present data.

This study has several strengths, including multi-timepoint sampling, paired assessment of circulating miRNAs, cytokines, and exosomes, and cross-comparison with an independent lung-cancer cohort. Nevertheless, important limitations should be acknowledged. These include modest sample sizes, the exploratory nature of the RNA-seq discovery phase, the observational study design, and the absence of mechanistic experiments, which together preclude causal inference regarding PM–miRNA relationships. In addition, predicted miRNA target pathways were not functionally validated; thus, pathway enrichment analyses should be interpreted as hypothesis-generating rather than confirmatory. Future studies employing controlled PM-exposure models, cell-type-specific miRNA profiling, and functional manipulation of candidate miRNAs (particularly miR-542-3p) will be necessary to determine whether these miRNAs act primarily as exposure biomarkers or as active mediators of PM-associated inflammatory and carcinogenic processes.

## 4. Materials and Methods

### 4.1. Identification of Candidate PM-Induced Plasma miRNA Through Small RNA Sequencing

This study was conducted in accordance with the principles of the Declaration of Helsinki. The research protocol, including participant recruitment, sample collection, and data handling procedures, was reviewed and approved by the Human Ethics Committee of the Faculty of Associated Medical Sciences, Chiang Mai University (Ethics Committee Name: Human Ethics Committee of the Faculty of Associated Medical Sciences, Chiang Mai University), Approval Code: AMSEC-66EX-004, Approval Date: 27 February 2023. Written informed consent was obtained from all participants prior to enrollment.

Plasma samples were collected from five healthy volunteers residing in Hang Dong district, Chiang Mai, Thailand during two distinct seasonal periods: the low particulate matter (PM) season in October (24 h mean < 15 µg/m^3^) and the high PM season in April (24 h mean > 35 µg/m^3^). These periods corresponded to the rainy season with cleaner air and the end of the dry-burning period with peak PM levels. Each participant provided blood samples in both seasons, allowing paired intra-individual comparisons.

Eligible participants were adults aged 35–60 years who had resided in Chiang Mai for at least 12 months and had no history or current diagnosis of cancer. Individuals were excluded if they had chronic respiratory disease (e.g., COPD, asthma), cardiovascular conditions, acute respiratory infection or febrile illness within two weeks prior to sampling, occupational exposure to elevated air pollutants beyond ambient environmental levels, or current pregnancy. For each collection, 5–10 mL of peripheral blood was drawn into EDTA tubes and processed within two hours. Plasma was separated by centrifugation, aliquoted, and stored at −80 °C until analysis. Total circulating miRNA was extracted using the NucleoSpin® miRNA Plasma Kit (MACHEREY-NAGEL, Düren, Germany) according to the manufacturer’s instructions.

Small RNA sequencing (small RNA-seq) was employed to comprehensively profile plasma miRNAs. In this study, ~10 ng of total small RNA from each sample was submitted to NovogeneAIT Genomics Singapore for library preparation and sequencing using an Illumina-compatible protocol. Small RNA libraries were prepared by ligating 3′ and 5′ adapters to small RNA fragments, followed by reverse transcription and PCR amplification with barcoded primers. Size selection was performed to enrich fragments corresponding to mature miRNAs (approximately 18–30 nucleotides). Libraries were sequenced on an Illumina platform to generate single-end reads, with a target depth sufficient to reliably detect low-abundance circulating miRNAs. Sequencing generated single-end reads at sufficient depth to detect low-abundance miRNAs. Primary data processing included adapter trimming and quality filtering with Trimmomatic v0.30 (reads with an average quality score < 20 and length < 18 nt were discarded). Clean reads were then analyzed with miRDeep2, which aligned reads to the human reference genome, annotated known miRNAs against miRBase, and predicted novel miRNAs based on characteristic hairpin structures. Novel miRNAs were defined according to miRDeep2 prediction criteria, including the presence of a characteristic hairpin secondary structure, significant read support for both mature and star sequences, and absence of annotation in miRBase. Only novel miRNA candidates detected consistently across samples and passing miRDeep2 confidence thresholds were retained for downstream analysis. Normalized expression values were generated for each detected miRNA. Differential expression analysis was performed using the DESeq2 Bioconductor package to compare miRNA profiles between low- and high-PM exposure periods within the same individuals. Significantly dysregulated miRNAs were identified and prioritized as candidate biomarkers for downstream validation and functional studies.

### 4.2. Particulate Matter (PM2.5) Measurement Using DUSTBOY

Ambient particulate levels were quantified using the DUSTBOY community air-quality network operating in Chiang Mai, Thailand. We obtained the continuous PM2.5 time series from the San Na Meng station (dashboard ID DBBlack232), which uses laser light-scattering optical particle counting to measure size-resolved particle number and converts these counts to mass (µg/m^3^) via manufacturer algorithms. Raw data covering the entire observation period (pre-haze, within-season rise, and peak haze) were downloaded at their native temporal resolution (minute/hour), converted to local time (UTC+7), and screened by a predefined quality-assurance protocol: negative or missing values were removed; hours coinciding with instrument resets or implausible spikes were flagged by distributional checks (upper 99.5th percentile and >3× IQR within month) and excluded only when accompanied by data dropouts; days with fewer than 18 valid hourly observations were treated as missing. Because optical measurements are sensitive to hygroscopic growth at high relative humidity, we conducted a sensitivity recalculation that down-weighted hours with RH > 85% using concurrent meteorological records; conclusions were unchanged, and the primary analysis uses the network’s calibrated series when available. For exposure characterization and figures, we computed daily 24 h means and short-term moving averages (3-, 7-, and 14-day windows) as well as monthly means to depict the seasonal biomass-burning haze. When multiple DUSTBOY stations were intermittently available, the station geographically closest to the study catchment was used; otherwise, San Na Meng served as the area-level proxy for ambient PM2.5.

### 4.3. Assessment of Candidate miRNA Expression Level in Plasma of Healthy Volunteers and Diabetes Patients

Blood samples were collected from residents of San Na Meng, San Sai District, Chiang Mai, including 28 individuals with type 2 diabetes and 29 age- and weight-matched non-diabetic controls. Each participant provided specimens at three defined timepoints: (i) the early phase of the PM season, (ii) after 3-4 consecutive months of sustained PM exposure, and (iii) one year after exposure when the baseline PM level was resumed. Participants with diabetes were aged ≥60 years, weighed ≥45 kg, and were diagnosed with type 2 diabetes according to the American Diabetes Association Standards of Care. Control participants were of similar age and weight, had no history of diabetes, and demonstrated fasting blood glucose levels < 100 mg/dL. Exclusion criteria applied to both groups and included imprisonment under prosecution; a history of autoimmune disease, AIDS, or organ transplantation; acute illness such as diarrhea within 7 days or symptoms of respiratory infection; unexplained weight loss within 3 months; major surgery within 6 months or minor surgery within 1 month; recent drug or alcohol use within 24 h; and vaccination within the previous 3 months. The research protocol was reviewed and approved by the Ethics Committee of the Faculty of Associated Medical Sciences, Chiang Mai University (Ethics Committee Name: Human Ethics Committee, Faculty of Associated Medical Sciences, Chiang Mai University; Approval Code: AMSEC-63EX-036; Approval Date: 22 October 2022). For each collection, venous blood (5–10 mL) was drawn into EDTA tubes, processed within 2 h, and centrifuged to separate plasma. Plasma aliquots were stored at −80 °C until analysis. Total circulating miRNA was isolated from plasma using the NucleoSpin^®^ miRNA Plasma Mini Kit (MACHEREY-NAGEL) following the manufacturer’s instructions, which included lysis of plasma proteins, ethanol precipitation, column-based purification, multiple wash steps, and elution in RNase-free water. RNA concentration and purity were assessed using a BioTek Eon™ Microplate Spectrophotometer (BioTek Instruments, Winooski, VT, USA).

Candidate miRNAs identified from small RNA sequencing were validated by RT-qPCR. Approximately 10 ng of RNA per reaction underwent poly(A) tailing and cDNA synthesis using the Tetro cDNA Synthesis Kit (Meridian Bioscience, Cincinnati, OH, USA). Quantitative PCR was performed using the iTaq™ Universal SYBR^®^ Green Supermix (Bio-Rad, Hercules, CA, USA) on a CFX96 Touch™ Real-Time PCR Detection System (Bio-Rad). Forward primers specific to each mature miRNA were designed using the sRNA PrimerDB database, and a universal reverse primer complementary to the adapter sequence was applied. miR-484, previously reported to have stable expression across plasma samples [42], was used as the endogenous control. Each reaction was run in duplicate, and no-template controls were included to monitor contamination. Amplification specificity was confirmed by melt-curve analysis. Relative expression levels were calculated using the 2^−ΔCT^ method, where ΔCT = CT(miRNA of interest) − CT(miR-484), and miRNAs showing statistically significant expression differences between groups were prioritized for downstream exosome analysis.

### 4.4. Exosome Extraction and Characterization

Exosomes were precipitated from plasma using a polymer-based reagent (Total Exosome Isolation Reagent, Thermo Fisher Scientific, Waltham, MA, USA)) according to the manufacturer’s protocol. Briefly, 250–500 µL of plasma was mixed with the reagent at a 2:1 ratio (*v*/*v*) and incubated overnight at 2–8 °C to promote vesicle precipitation. Samples were centrifuged at 10,000× *g* for 60 min at 4 °C, and the resulting exosomal pellet was resuspended in phosphate-buffered saline (PBS). All samples were processed using the same protocol, reagent lot, incubation conditions, and centrifugation parameters to ensure comparability across participants and timepoints. Resuspended vesicles were either processed immediately for downstream applications or stored at −80 °C until further analysis to preserve vesicle integrity.

Polymer-based precipitation was selected for this exploratory human study because it is compatible with limited plasma volumes and enables consistent recovery for downstream particle characterization. Exosome characterization was performed using VideoDrop Nanoparticle Tracking Analysis (NTA) and Transmission Electron Microscopy (TEM). For NTA, 10 µL of resuspended exosomes was diluted in 990 µL of PBS to achieve a particle concentration within the optimal detection range (about 10^7^–10^9^ particles/mL). Each sample was analyzed in triplicate, with three 60 s videos recorded per replicate. Brownian motion of particles was tracked under laser illumination, the diffusion coefficient was calculated, and particle diameters were derived using the Stokes–Einstein equation. This provided both size distribution and vesicle concentration. For NTA, identical instrument settings (camera level and detection threshold) were applied across all runs within the same experiment.

For TEM, exosome suspensions were fixed in 2% paraformaldehyde and applied to formvar/carbon-coated copper grids. After 20 min of adsorption, grids were washed with PBS and negatively stained with 2% uranyl acetate. The grids were air-dried and examined using a transmission electron microscope operated at 80–120 kV. Exosomes were visualized as round or cup-shaped vesicles with diameters in the expected range of 30–200 nm, confirming both morphology and structural integrity. We note that polymer-based isolation may co-precipitate non-vesicular components; however, all samples were processed identically, enabling valid relative comparisons between groups and timepoints.

### 4.5. Plasma Cytokine Assessment

EDTA blood was processed within 2 h (≈1500–2000× *g* centrifugation, 10 min, 4 °C); plasma was aliquoted and stored at −80 °C (single freeze–thaw). IL-6, IL-8, and TNF-α were quantified by sandwich ELISA using BD OptEIA™ Human ELISA Sets—IL-6 (Cat. 555220), IL-8 (Cat. 555244), and TNF (Cat. 555212) (BD Biosciences, San Diego, CA, USA) according to the manufacturer’s protocols. High-binding 96-well plates were coated overnight at 4 °C with capture monoclonal antibody, blocked, and loaded with standards and appropriately diluted samples in duplicate. After incubation with the biotinylated detection monoclonal antibody and streptavidin–HRP, signal was developed with TMB, stopped with acid, and read at 450 nm with 570 nm reference. A 7-point standard curve was run on each plate and fit by four-parameter logistic (4-PL) regression; concentrations were interpolated and multiplied by dilution factors. Assays are calibrated to WHO/NIBSC materials (IL-6: 89/548, conversion 1 µg NIBSC = 1.44 µg BD rhIL-6; IL-8: 89/520, 1 µg = 1.06 µg BD rhIL-8; TNF: 87/650, 1 µg = 1.14 µg BD rhTNF). Runs were accepted when duplicate %CV ≤ 15%; out-of-range samples were re-assayed at appropriate dilutions.

### 4.6. Comparison of PM-Responsive miRNAs in Healthy Controls and Lung-Cancer Patients

To assess the clinical relevance of PM-responsive miRNAs, we compared the circulating levels of four key candidates—miR-542-3p, miR-29a-3p, novelmiR-203, and novelmiR-754—between healthy individuals and patients with lung cancer. Previously collected, de-identified serum samples from 27 healthy volunteers and 55 newly diagnosed, treatment-naïve lung-cancer patients attending Maharaj Nakorn Chiang Mai Hospital were analyzed. All participants had provided written informed consent at the time of collection, and the protocol was approved by “the Human Ethics Committee of the Faculty of Medicine, Chiang Mai University” (Approval Number: NONE-2564-08207, Approval Date: 25 May 2021).

For each participant, venous blood (5–10 mL) was collected into serum separator tubes, allowed to clot at room temperature, and centrifuged within 2 h to obtain serum. Aliquots were stored at −80 °C until analysis. Total circulating miRNA was extracted using the NucleoSpin^®^ miRNA Plasma Mini Kit (MACHEREY-NAGEL) according to the manufacturer’s instructions. cDNA synthesis was performed with the Tetro cDNA Synthesis Kit (Meridian Bioscience), followed by RT-qPCR using iTaq™ Universal SYBR^®^ Green Supermix (Bio-Rad) on a CFX96 Touch™ Real-Time PCR Detection System (Bio-Rad). Forward primers specific for miR-542-3p, miR-29a-3p, novelmiR-203, and novelmiR-754 were designed based on the mature sequences, together with the universal reverse primer described in Section 4.3. miR-484 was used as the endogenous reference. Amplification specificity was verified by melt-curve analysis. Relative expression levels were calculated using the 2^−ΔCT^ method, and group differences between healthy controls and lung-cancer patients were evaluated using the Mann–Whitney U test, with *p* < 0.05 considered statistically significant.

## 5. Conclusions

Seasonal particulate matter exposure in Northern Thailand was associated with changes in circulating miRNAs, IL-8, and exosome profiles, with distinct response patterns in adults with and without diabetes. Using a stepwise discovery and validation framework, miR-542-3p and miR-29a-3p emerged as candidate PM-responsive circulating miRNAs, and diabetes appeared to modify both baseline levels and seasonal responsiveness. Reduced circulating miR-542-3p in peak-exposure periods and in the lung-cancer cohort supports potential clinical relevance of this marker.

Given the modest sample sizes and observational design, these findings should be interpreted as associative and hypothesis-generating. Larger longitudinal studies and mechanistic work are needed to validate these miRNAs as exposure/susceptibility biomarkers and clarify their functional roles.

## Figures and Tables

**Table 1:** Differentially expressed circulating miRNAs in high- versus low-PM samples (discovery cohort; n = 5 per group).

miRNA	log_2_FC	*p*-Value	FDR (padj)	Regulation
hsa-miR-542-3p	−24.4	<0.001	<0.001	Down
hsa-miR-370-3p	−5.6	0.05	0.70	Down
hsa-miR-2115-5p	−6.3	0.04	0.70	Down
hsa-let-7d-3p	−6.2	0.03	0.70	Down
hsa-miR-1292-5p	−6.6	0.03	0.70	Down
novelmiR-165 5′-uugaggucggacaugguggcu-3′	−6.6	0.03	0.70	Down
hsa-miR-493-3p	−6.8	0.02	0.70	Down
novelmiR-886 5′-auacugggauauuuggagcuuc-3′	−7.0	0.02	0.70	Down
hsa-miR-27a-3p	6.0	0.01	0.70	Up
novelmiR-734 -3′ugugugucuguaucucuuc-3′	6.1	0.05	0.70	Up
hsa-miR-29a-3p	24.2	<0.001	<0.001	Up
novelmiR-754 5′-uaguggucucuguaucucugggaag-3′	26.1	<0.001	<0.001	Up
novelmiR-203 5′-gggguggggucugaggauuuguga-3′	26.7	<0.001	<0.001	Up

List of 13 plasma miRNAs identified by small RNA sequencing as significantly dysregulated between individuals sampled in low- and high-PM environments (discovery cohort). Log_2_FC represents the log2 fold change in expression in high-PM relative to low-PM samples (negative values indicate downregulation). *p*-values and FDR (padj) were obtained from DESeq2 Wald tests, and “Regulation” denotes direction of change in the high-PM group. These miRNAs comprise the PM-responsive signature used for downstream GO enrichment and longitudinal validation analyses.

**Table 2:** Baseline demographic and clinical characteristics of non-diabetes (non-DM) and diabetes (DM) participants included in the longitudinal miRNA analysis.

Variable	Healthy (Mean CSD)	Diabetes (Mean ± SD)	*p* Value *
Sample size (n)	29	28	
Age (years)	69.3 ± 6.49	68.6 ± 5.88	0.581 ^a^
Sex			0.256 ^b^
Male Female	8 21	11 19	
Weight (kg)	57.4 ± 10.95	63.1 ± 7.97	0.031 ^a,^*
Height (cm)	151.3 ± 8.01	153.8 ± 6.81	0.212 ^a^
BMI (kg/m^2^)	25.0 ± 4.35	26.7 ± 2.94	0.108 ^a^
Systolic BP (mmHg)	135.5 ± 18.13	134.8 ± 19.02	0.886 ^a^
Diastolic BP (mmHg)	70.7 ± 11.59	69.7 ± 10.58	0.746 ^a^
SpO_2_ (%)	97.8 ± 0.58	97.7 ± 0.70	0.613 ^a^
Pulse (bpm)	83.2 ± 12.49	86.3 ± 13.72	0.376 ^a^
hsCRP (mg/L)	2.22 ± 3.316	1.73 ± 1.758	0.496 ^a^
FBS (mg/dL)	108 ± 13.6	103 ± 7.786	0.087 ^a^
HbA1c (%)	5.7 ± 0.27	5.3 ± 0.62	0.003 ^a,^*

^a^ Unpaired *t*-test, ^b^ Chi-square, * statistically different at *p* < 0.05. Data represent mean ± SD. Group differences were assessed using unpaired *t*-tests for continuous variables and chi-square tests for categorical variables. HbA1c, weight, and selected metabolic parameters differed significantly between groups, consistent with expected clinical features of diabetes, whereas age, sex distribution, anthropometric measures, and hemodynamic variables were comparable. * *p* < 0.05 was considered statistically significant.

**Table 3:** Demographic and clinical characteristics of lung-cancer patients and healthy controls included in the cross-sectional miRNA comparison.

Variable	Lung Cancer	Healthy Controls
Sample size (n)	55	27
Age (years)	65.7 ± 11.55	62.4 ± 8.44
Gender		
Male	29	12
Female	26	15
Smoking Status		
Smokers	34	1
Non-smokers	21	26
Histology		
Adenocarcinoma Squamous cell carcinoma	44 11	Not applicable
Stage		
Early (Stage I and II) Late (Satge III and IV)	15 40	Not applicable

## Data Availability

Data are available from the corresponding author on reasonable request.

## Abbreviations

The following abbreviations are used in this manuscript:

AKT	Protein kinase B
AML	Acute myeloid leukemia
ARHGAP1	Rho GTPase-activating protein 1
BMI	Body mass index
BP	Blood pressure
CRP	C-reactive protein
CXCR1/2	C-X-C motif chemokine receptor 1/2
DESeq2	Differential Expression Sequencing 2
DM	Diabetes mellitus
EDTA	Ethylenediaminetetraacetic acid
ELISA	Enzyme-linked immunosorbent assay
ER	Endoplasmic reticulum
FBS	Fasting blood sugar
FDR	False discovery rate
GO	Gene Ontology
HbA1c	Glycated hemoglobin
HRP	Horseradish peroxidase
hsCRP	High-sensitivity C-reactive protein
IL-6	Interleukin-6
IL-8	Interleukin-8
IQR	Interquartile range
miRNA/miRNAs	microRNA(s)
M0	Baseline (early PM season) sampling timepoint
M3	Post-exposure (after 3-4 months of sustained high PM season) sampling timepoint
NF-κB	Nuclear factor kappa-light-chain-enhancer of activated B cells
NTA	Nanoparticle tracking analysis
PBS	Phosphate-buffered saline
PCR	Polymerase chain reaction
PM	Particulate matter
PM2.5	Particulate matter ≤ 2.5 µm in aerodynamic diameter
PM10	Particulate matter ≤ 10 µm in aerodynamic diameter
ROS	Reactive oxygen species
RT-qPCR	Reverse transcription quantitative polymerase chain reaction
TEM	Transmission electron microscopy
TNF-α	Tumor necrosis factor-alpha
WHO	World Health Organization
Y1	Follow-up sampling timepoint (starting of next PM season)

## References

[1] Kawichai S., Sripan P., Rerkasem A., Rerkasem K., Srisukkham W. (2025). Long-Term Retrospective Predicted Concentration of PM(2.5) in Upper Northern Thailand Using Machine Learning Models. Toxics.

[2] Saksakulkrai S., Chantara S., Kraisitnitikul P., Srivastava D., Shi Z. (2026). Unveiling the origins of Northern Thailand’s haze: Comprehensive chemical characterization and source apportionment of PM(2.5) using targeted molecular markers. J. Environ. Sci..

[3] Ngamsang P., Amnuaylojaroen T., Parasin N., Pimonsree S. (2023). Health Impact Assessment of Short-Term Exposure to Particulate Matter (PM(10)) in Northern Thailand. J. Environ. Public Health.

[4] Sapbamrer P., Assavanopakun P., Panumasvivat J. (2024). Decadal Trends in Ambient Air Pollutants and Their Association with COPD and Lung Cancer in Upper Northern Thailand: 2013–2022. Toxics.

[5] Wang J., Huang J., Wang L., Chen C., Yang D., Jin M., Bai C., Song Y. (2017). Urban particulate matter triggers lung inflammation via the ROS-MAPK-NF-kappaB signaling pathway. J. Thorac. Dis..

[6] Yan Z., Wang J., Li J., Jiang N., Zhang R., Yang W., Yao W., Wu W. (2016). Oxidative stress and endocytosis are involved in upregulation of interleukin-8 expression in airway cells exposed to PM2.5. Environ. Toxicol..

[7] Chen Y., Yan A., Zhang L., Hu X., Chen L., Cui J., Fan Z., Li Y. (2025). Comparative analysis of inflammatory biomarkers for the diagnosis of neonatal sepsis: IL-6, IL-8, SAA, CRP, and PCT. Open Life Sci..

[8] Iskandar A., Mayashinta D.K., Robert R., Samsu N., Endharti A.T., Widjajanto E. (2023). Correlation Between IL-8, C-Reactive Proteins (CRP) and Neutrophil to Lymphocyte Ratio (NLR) as Predictor of Mortality in COVID-19 Patients with Diabetes Mellitus Comorbidity. Int. J. Gen. Med..

[9] Schneider A., Alexis N.E., Diaz-Sanchez D., Neas L.M., Harder S., Herbst M.C., Cascio W.E., Buse J.B., Peters A., Devlin R.B. (2011). Ambient PM2.5 exposure up-regulates the expression of costimulatory receptors on circulating monocytes in diabetic individuals. Environ. Health Perspect..

[10] Chen X., Han Y., Chen W., Wang Y., Qiu X., Li W., Hu M., Wu Y., Wang Q., Zhang H. (2020). Respiratory Inflammation and Short-Term Ambient Air Pollution Exposures in Adult Beijing Residents with and without Prediabetes: A Panel Study. Environ. Health Perspect..

[11] Liu C., Bai Y., Xu X., Sun L., Wang A., Wang T.Y., Maurya S.K., Periasamy M., Morishita M., Harkema J. (2014). Exaggerated effects of particulate matter air pollution in genetic type II diabetes mellitus. Part. Fibre Toxicol..

[12] Khafaie M.A., Salvi S.S., Ojha A., Khafaie B., Gore S.D., Yajnik C.S. (2018). Particulate matter and markers of glycemic control and insulin resistance in type 2 diabetic patients: Result from Wellcome Trust Genetic study. J. Expo. Sci. Environ. Epidemiol..

[13] Lao X.Q., Guo C., Chang L.Y., Bo Y., Zhang Z., Chuang Y.C., Jiang W.K., Lin C., Tam T., Lau A.K.H. (2019). Long-term exposure to ambient fine particulate matter (PM(2.5)) and incident type 2 diabetes: A longitudinal cohort study. Diabetologia.

[14] Liu T., Chen X., Xu Y., Wu W., Tang W., Chen Z., Ji G., Peng J., Jiang Q., Xiao J. (2019). Gut microbiota partially mediates the effects of fine particulate matter on type 2 diabetes: Evidence from a population-based epidemiological study. Environ. Int..

[15] Guo J., Xie X., Wu J., Yang L., Ruan Q., Xu X., Wei D., Wen Y., Wang T., Hu Y. (2022). Association between fine particulate matter and coronary heart disease: A miRNA microarray analysis. Environ. Pollut..

[16] Li X., Haberzettl P., Conklin D.J., Bhatnagar A., Rouchka E.C., Zhang M., O’Toole T.E. (2021). Exposure to Fine Particulate Matter Air Pollution Alters mRNA and miRNA Expression in Bone Marrow-Derived Endothelial Progenitor Cells from Mice. Genes.

[17] Liu X., Chai B., Wang X., Wu Z., Zou H., Liu Y., Zheng S., Qian G., Ma Z., Lu J. (2024). Environmentally Persistent Free Radical Promotes Lung Cancer Progression by Regulating the Expression Profile of miRNAs. Cancer Biother. Radiopharm..

[18] Chen Z., Ji N., Wang Z., Wu C., Sun Z., Li Y., Hu F., Wang Z., Huang M., Zhang M. (2018). Fine Particulate Matter (PM(2.5)) Promoted the Invasion of Lung Cancer Cells via an ARNT2/PP2A/STAT3/MMP2 Pathway. J. Biomed. Nanotechnol..

[19] Colin-Val Z., Flores-Navarro G., Rocha-Zavaleta L., Robledo-Cadena D.X., Quintana-Belmares R.O., Lopez-Marure R. (2024). Fine particulate matter (PM(2.5)) promotes chemoresistance and aggressive phenotype of A549 lung cancer cells. Toxicol. Appl. Pharmacol..

[20] Wei J., Li F., Yang J., Liu X., Cho W.C. (2015). MicroRNAs as regulators of airborne pollution-induced lung inflammation and carcinogenesis. Arch. Toxicol..

[21] Liao D., Wei S., Hu J. (2024). Inhibition of miR-542-3p augments autophagy to promote diabetic corneal wound healing. Eye Vis (Lond).

[22] Yin Q., Guo N., Liao R. (2025). LncRNA GAS5 reduces blood glucose levels and alleviates renal fibrosis in diabetic nephropathy by regulating the miR-542-3p/ERBB4 axis. Diabetol. Metab. Syndr..

[23] Qasim Q. (2024). Antioxidants, Lipid Profiles, and Glucose Levels, as Well as Persistent Inflammation, Are Central to the Link between Diabetes Mellitus Type Ii and Oxidative Stress. Georgian Med. News.

[24] Weinberg Sibony R., Segev O., Dor S., Raz I. (2024). Overview of oxidative stress and inflammation in diabetes. J. Diabetes.

[25] Alshahrani S.H., Rakhimov N., Gupta J., Hassan Z.F., Alsalamy A., Saleh E.A.M., Alsaab H.O., Al-Aboudy F.K., Alawadi A.R., Mustafa Y.F. (2023). The mechanisms, functions and clinical applications of miR-542-3p in human cancers. Pathol. Res. Pract..

[26] Liu B., Li J., Zheng M., Ge J., Li J., Yu P. (2017). MiR-542-3p exerts tumor suppressive functions in non-small cell lung cancer cells by upregulating FTSJ2. Life Sci..

[27] Althoff K., Lindner S., Odersky A., Mestdagh P., Beckers A., Karczewski S., Molenaar J.J., Bohrer A., Knauer S., Speleman F. (2015). miR-542-3p exerts tumor suppressive functions in neuroblastoma by downregulating Survivin. Int. J. Cancer.

[28] Chen X., Jin Y., Feng Y. (2019). Evaluation of Plasma Extracellular Vesicle MicroRNA Signatures for Lung Adenocarcinoma and Granuloma with Monte-Carlo Feature Selection Method. Front. Genet..

[29] Gado M.M., Mousa N.O., Badawy M.A., El Taweel M.A., Osman A. (2019). Assessment of the Diagnostic Potential of miR-29a-3p and miR-92a-3p as Circulatory Biomarkers in Acute Myeloid Leukemia. Asian Pac. J. Cancer Prev..

[30] Shi T., Wu X., Liu A. (2025). Upregulated miR-29a-3p Prevent Malignant Features of Lymphoma Cells by Targeting MCL1. Hematol. Oncol..

[31] Fellizar A., Refuerzo V., Ramos J.D., Albano P.M. (2023). Expression of specific microRNAs in tissue and plasma in colorectal cancer. J. Pathol. Transl. Med..

[32] Yamada A., Horimatsu T., Okugawa Y., Nishida N., Honjo H., Ida H., Kou T., Kusaka T., Sasaki Y., Yagi M. (2015). Serum miR-21, miR-29a, and miR-125b Are Promising Biomarkers for the Early Detection of Colorectal Neoplasia. Clin. Cancer Res..

[33] Wen Q., Wang Y., Li X., Jin X., Wang G. (2021). Decreased serum exosomal miR-29a expression and its clinical significance in papillary thyroid carcinoma. J. Clin. Lab. Anal..

[34] Yamada H., Suzuki K., Fujii R., Kawado M., Hashimoto S., Watanabe Y., Iso H., Fujino Y., Wakai K., Tamakoshi A. (2021). Circulating miR-21, miR-29a, and miR-126 are associated with premature death risk due to cancer and cardiovascular disease: The JACC Study. Sci. Rep..

[35] Dinh T.K., Fendler W., Chalubinska-Fendler J., Acharya S.S., O’Leary C., Deraska P.V., D’Andrea A.D., Chowdhury D., Kozono D. (2016). Circulating miR-29a and miR-150 correlate with delivered dose during thoracic radiation therapy for non-small cell lung cancer. Radiat. Oncol..

[36] Chioccioli M., Roy S., Newell R., Pestano L., Dickinson B., Rigby K., Herazo-Maya J., Jenkins G., Ian S., Saini G. (2022). A lung targeted miR-29 mimic as a therapy for pulmonary fibrosis. EBioMedicine.

[37] Cushing L., Kuang P., Lu J. (2015). The role of miR-29 in pulmonary fibrosis. Biochem. Cell Biol..

[38] Kurai J., Onuma K., Sano H., Okada F., Watanabe M. (2018). Ozone augments interleukin-8 production induced by ambient particulate matter. Genes. Environ..

[39] Xiong X., Liao X., Qiu S., Xu H., Zhang S., Wang S., Ai J., Yang L. (2022). CXCL8 in Tumor Biology and Its Implications for Clinical Translation. Front. Mol. Biosci..

[40] Eckhardt C.M., Baccarelli A.A., Wu H. (2022). Environmental Exposures and Extracellular Vesicles: Indicators of Systemic Effects and Human Disease. Curr. Environ. Health Rep..

[41] Xu H., Jiao X., Wu Y., Li S., Cao L., Dong L. (2019). Exosomes derived from PM2.5-treated lung cancer cells promote the growth of lung cancer via the Wnt3a/beta-catenin pathway. Oncol. Rep..

[42] Liu Y.J., Wang C. (2023). A review of the regulatory mechanisms of extracellular vesicles-mediated intercellular communication. Cell Commun. Signal.

